# Button Gastrostomy Tubes for Pediatric Patients: A Tertiary Care Center Experience

**DOI:** 10.1155/2020/5286283

**Published:** 2020-10-08

**Authors:** Fayza Haider, Hasan Mohamed Ali Isa, Mohamed Amin Al Awadhi, Barrak Ayoub, Ezat Bakhsh, Husain Al Aradi, Shahraban Abdulla Juma

**Affiliations:** ^1^Department of Surgery-Pediatric Surgery Unit, Clinical Lecturer, Arabian Gulf University, Manama, Bahrain; ^2^Pediatric Department, Salmaniya Medical Complex, Assistant Professor, Arabian Gulf University, Manama, Bahrain; ^3^Department of Surgery-Pediatric Surgery Unit, Assistant Professor, Arabian Gulf University, Manama, Bahrain; ^4^Department of Surgery-Pediatric Surgery Unit, Salmaniya Medical Complex, Manama, Bahrain; ^5^Department of Surgery, Salmaniya Medical Complex, Manama, Bahrain

## Abstract

**Results:**

Out of 34 patients who underwent gastrostomy tube insertion, 30 patients had their long tube replaced by a button gastrostomy. Majority were males (*N* = 18, 60%). Prolonged nasogastric tube feeding was the main indication of referral (*N* = 17, 56%) followed by feed intolerance (*N* = 6, 17%) and gastroesophageal reflux disease (*N* = 5, 16%). The main underlying diseases at referral were neurological impairment (*N* = 19, 63%) and metabolic disorders (*N* = 4, 13%). There was no significant difference between patients with neurological disorders and other diseases in terms of gender, nationality, or age. Laparotomy with gastrostomy is the main approach used (*N* = 18, 60%). No reported complications of button tubes in 50% of the patients (*N* = 15).

**Conclusions:**

Prolonged nasogastric tube feeding is the main indication of referral for gastrostomy tube insertion. Neurological disorders are the main diagnosis for the cases operated upon. Laparotomy with gastrostomy is the procedure of choice at our center. Majority of patients had no reported complications of button tube replacement. These children are likely to benefit from the button tube with fewer complications.

## 1. Introduction

In children who are not able to be nourished by mouth, other routes must be sought. If this is a short temporary condition, nasogastric tube routes are feasible, but long-term use increases the risk of complications [1].

A gastrostomy is a surgical opening through the abdomen into the stomach where a feeding device is inserted. Gastrostomy tubes do not cause irritation to the nasal mucosa, facial skin, or the esophagus. They reduce the risk of tube displacement and have less risk of pulmonary aspiration, frequent reinsertion, and interruptions to feeding.

Gastrostomy tube insertion is one of the most common procedures performed in the pediatric age group as a radical choice to overcome feeding difficulty, according to the European Society for Clinical Nutrition and Metabolism. Gastrostomy tube should be inserted for all patients who face feeding difficulty for more than 2-3 weeks [2, 3]. It appears to be reported more frequently in certain populations, such as those with neurological impairment, gastrointestinal tract (GIT) abnormalities, and cardiac defects [4].

When discussing gastrostomy tubes (G-tubes), it is important to specify the type of tube used. It can be a long G-tube like a Malecot or even a Foleys catheter, percutaneous endoscopic gastrostomy (PEG) tube, or a low-profile tube or “button”. The most common type of button is called a MIC-KEY (although a recent advance has been an even lower-profile tube called a MINI-ONE) [5]. Buttons are easily managed and less likely to get dislodged. Their local care is easier and has a better cosmetic appearance compared to the long tubes. All these devices are placed through a gastrostomy.

Salmaniya Medical Complex (SMC) is the only tertiary health care institute in the Kingdom of Bahrain, and it is the only institution offering button gastrostomy tubes. The replacement of a button tube instead of a long tube in the stomach for feeding is the practice in our hospital that was introduced in 2009. The replacement was initiated by the unit to follow the best practice internationally; this was thoroughly discussed with parents of patients who had the initial replacement and thereafter it became the procedure of choice. There are no reported data regarding pediatric patients with gastrostomies in the Kingdom of Bahrain. Hence, the purpose of this study was therefore to identify the medical conditions and indications associated with gastrostomy placement and outcome of replacement of button gastrostomy tubes instead of long tubes.

## 2. Subjects and Methods

### 2.1. Study Participants

A retrospective cross-sectional descriptive review of medical records (both chart review and electronic health records) at SMC, Kingdom of Bahrain, was conducted between January 2009 and August 2019. Pediatric age group patients from birth to 14 years of age who underwent button tube replacement were included in the study.

Patients' list was obtained by two methods and was compared and finalized:

1. Button gastrostomy tube registry in the pediatric surgery ward since 2009
2. List of all diagnoses of gastrostomy since 2009 from the medical records for ages 0 to 14 years. Using Classification of Surgical Operations and Procedures (OPCS) (4^th^ revision), OPCS-4 Code G34.1-G34.9

All patients found in the list were included in the study. Patients' charts were extracted from the Medical Records Department, and patient's data were collected by the Pediatric Surgery Team.

### 2.2. Data Collection

Participants' records were identified. A data collection sheet was constructed, and an Excel sheet was formed for the data to be entered immediately during collection. Basic demographic data including date of birth, gender, age, and diagnosis were collected. Indications for referral like feed intolerance, weight gain, motility disorders, severe gastroesophageal reflux disease, metabolic diseases, neurological disorders, and others were also gathered. These were later categorized for ease of analysis. Surgical information such as the center where the procedure was performed either in Bahrain (SMC) or out of the region, date of admission, date of surgery, date of discharge, length of stay (in days), type of procedures performed (laparotomy with gastrostomy alone, laparotomy with fundoplication and gastrostomy, Percutaneous Endoscopic Gastrostomy [PEG], or gastrostomy via interventional radiology) were noted. Surgical complications like wound infection, granulation around the opening, tube dislodgment, stenosis, closure due to tube incidentally being removed or pulled out, leak, tube rupture, intestinal obstruction, and others were also collected. The follow-up period was calculated based on the date of the last outpatient clinic visit.

### 2.3. Statistical Analysis

The coded data were statistically analyzed using SPSS for Windows version 23. The frequency and percentage of the categorical variables were calculated. The continuous variables were presented as mean and standard deviation (SD) or median and range. The patients were divided into two groups based on the indications for the procedure (neurological indication and others). The two groups were compared in terms of gender, nationality, and age category. Fisher's Exact test was used. *P* value < 0.05 was considered as statistically significant. The confidence interval was set at 95%.

Where data were missing, the participants were excluded from the analysis of the specific variable.

### 2.4. Ethical Approval

This study was conducted in accordance with the principles of Helsinki Declaration. It was ethically approved by the secondary care medical research subcommittee, Salmaniya Medical Complex, Ministry of Health, Kingdom of Bahrain.

## 3. Results

During the study period, a total of 34 patients underwent gastrostomy tube insertion. Of which, 30 patients underwent Mickey gastrostomy tube replacement instead of the long gastrostomy tube. Eighteen (60%) patients were males and 12 (40%) were females. The median (range) age at presentation was five years (range 0-13 years). The patients were divided into two groups based on the indications for the procedure (neurological indication and others). The two groups were compared in terms of gender, nationality, and age category. Fisher's Exact test was used. *P* value < 0.05 was considered as statistically significant. The confidence interval was set at 95%. The results are shown in Table 1. The mean (± SD) age at the time of study was 11.9 (5.37) years old.

There was no significant difference between patients with neurological disorders and those with other diseases in terms of gender, nationality, or age category.

**Figure 1:**
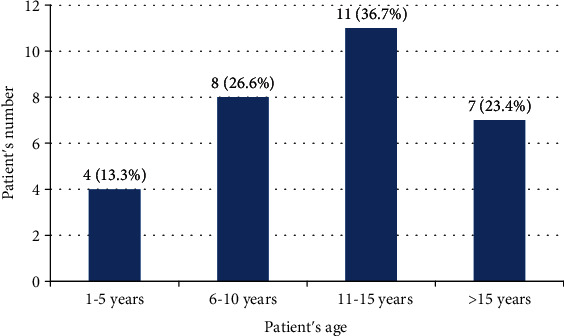
Age distribution of all patients that had a button gastrostomy tube.

The age distribution of the patients that had a button gastrostomy tube is shown in Figure 1. The highest number of patients is in the age group of 11-15 years, total of 11 patients (36.7%).

**Figure 2:**
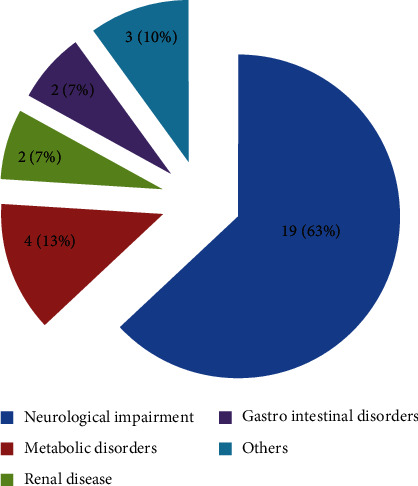
Patient's underlying disease at the time of referral.

The main underlying diseases at the time of referral were neurological impairment (*N* = 19, 63%) (cerebral palsy in 12 patients, anoxic brain injury in three, and encephalopathy, traumatic head injury, spina bifida, and aicardi syndrome each in one patient) followed by metabolic disorders (*N* = 4, 13%) (hypermelanosis, Niemann pick disease, Sanjad Sakati syndrome, and one unspecified metabolic disease) (Figure 2). Two patients had renal diseases (dysplastic kidney disease and chronic renal failure). Two had gastrointestinal disorders (Crohn's disease and cystic fibrosis). Three patients had other indications for referral (10%), one had congenital muscular dystrophy, one patient had Treacher Collin syndrome, and one had Trisomy 18 syndrome.

**Figure 3:**
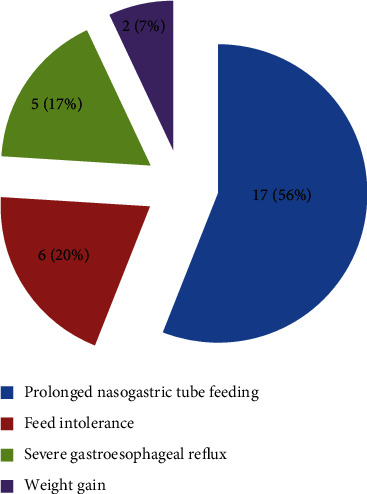
Reason for referral for gastrostomy tube insertion.

Prolonged nasogastric tube feeding was the main indication of referral for gastrostomy tube insertion (*N* = 17, 56%) followed by feed intolerance (*N* = 6, 20%), severe gastroesophageal reflux disease (*N* = 5, 17%), and for weight gain (*N* = 2, 7%) (Figure 3).

Twenty-four (80%) of the patients had available data about the place of procedure, 16 (66.7%) of them were operated on in our institution, and the remaining eight (33.3%) has been performed out of our center. Laparotomy and gastrostomy (Stamm) is the main approach used (*N* = 18, 60%), followed by laparotomy with fundoplication (*N* = 6, 20%) and Percutaneous Endoscopic Gastrostomy (PEG) (*N* = 6, 20%). One patient had his gastrostomy tube placed via interventional radiology that was later revised by the pediatric surgeon.

**Figure 4:**
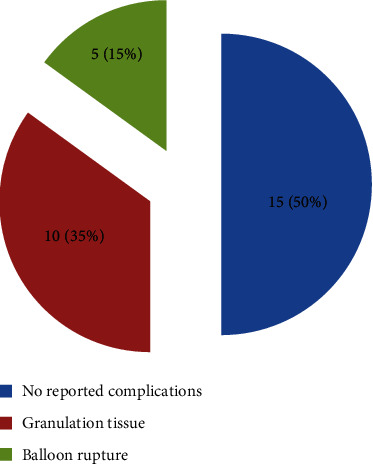
Complications associated with gastrostomy tube insertion.

Complications of button tube insertion are shown in Figure 4. No patient was complicated by any wound infection, tube dislodgment, stenosis, closure due to tube incidentally being removed or pulled out, leak, and intestinal obstruction. All patients who developed granulation tissue around the gastrostomy were treated with topical silver nitrate stick. The mean (standard deviation) of the length of stay (including the admission days in pediatric and pediatric surgery wards) was 9 ± 3.57 days. Twenty (70%) patients of the study population are still following up in pediatric surgery outpatient clinic. The rest were lost to follow-up in the clinic but parents do come to the ward for tube supply.

## 4. Discussion

Salmaniya Medical Complex is the first institution in Bahrain that offered pediatric surgery services. It still receives almost all referrals for gastrostomy tube insertion. Gastrostomy tube insertion is one of the most commonly performed operations in the practice of pediatric surgery [4]. Neurological impairment has been associated with gastrostomy placement in the pediatric population. Cerebral palsy was the main neurological diagnosis, where they frequently have dysphagia and therefore may require gastrostomy feeding [4].

The underlying diseases in our patients were similar as reported elsewhere [1], with the majority of our patients being diagnosed with neurological disorders (63%).

The placement of a gastrostomy tube in a pediatric patient often represents a seminal moment for the child, family, and medical team providing care. The implications of the procedure continue for years to follow as care plans shift to outpatient management and nutritional needs become definitively addressed. Ultimately, the technique chosen for gastrostomy tube placement should rest with the implicated health care team, individualized for each unique patient circumstance, and be based on individual/institutional expertise [6].

The technique of gastrostomy tube insertion has undergone several modifications throughout the past decades. It has evolved from the traditional Stamm procedure to PEG, and more recently, laparoscopic gastrostomy [7]. Stamm procedures were performed on 60% of our study population which is the procedure of choice at our institution. The PEG technique offered the advantage of scar-less operation with a shorter time in the operating room [3]. When performed in relatively uncomplicated infants or neonates where gastrostomy is the only procedure performed, PEG has similar risks of postoperative complications as compared to the surgical G-tube [8]. Once PEG was available in our center, 20% of the cases received this tube type insertion that was later removed and changed to button tube. The method of gastrostomy tube placement must be carefully chosen for each patient with specific attention to patient comorbidities, body habitus, and experience [9].

Gastrostomy placement is a common general pediatric surgical procedure that is usually straightforward and simple. However, major and minor complications can lead to a significant burden for the patients and their families, in the form of multiple clinic visits, readmissions, and even repeated surgical procedures [10].

The most common complications mentioned are leakage around the tube, wound or stoma infection, pneumoperitoneum, tract disruption with intraperitoneal spillage, and granulation [11]. Hypergranulation tissue was the most common postoperative complication, and tube dislodgement was the second most common complication according to Naiditch et al. [12].

The initially described Foley catheters have been demonstrated to have increased morbidity rate due to tube leakage, breakage, migration, proximal small bowel obstruction, and gastric wall penetration whereas gastrostomy buttons (balloon and mushroom types) consist of a low-profile catheter with a feeding hub, designed for mature tracts [13].

The replacement was initiated by the unit to follow the best practice internationally. Parents were given the choice to stay with the long tube or change to button tubes with thorough explanation of the indications and complications. This was started with parents of patients who had the initial replacement and thereafter it became the procedure of choice. Most of our study population (30/34) chose to change the long tubes to the button.

In our study, there were no reported complications in half of the study population, 35% had granulation tissue that was treated with silver nitrate stick [5], and 15% had balloon rupture.

## 5. Study Limitation

This study was limited by its retrospective nature and the relatively small sample size. Despite the limitations, the present study is the first in our country and may provide a starting point for specific information on gastrostomy placement in our pediatric population. This study would be a useful reference for future studies on this subject, as well as introducing this tube into our clinical practice. We aim at having it as the Benchmark study and a reference point in our country.

## 6. Conclusion

Prolonged use of a nasogastric feeding tube is the main indication of referral for gastrostomy tube insertion. Neurological disorders are the main diagnosis for the cases operated upon. Laparotomy with Stamm gastrostomy is the procedure of choice at our center. The majority of patients had no reported complications of button tube replacement. These children are likely to benefit from the button tube with fewer complications.

## Figures and Tables

**Table 1:** Comparison between the neurological indications for button gastrostomy tubes for 30 pediatric patients and other indications in term of gender, nationality, and age category.

Variable		Neurological indications *N* = 17 (56.7%)	Other indications^∗^*N* = 13 (43.3%)	*P* value
Gender	Male	10 (58.8)	8 (61.5)	0.590^†^
	Female	7 (41.2)	5 (38.5)	
Nationality	Bahraini	17 (100)	12 (92.3)	0.433^†^
	Non-Bahraini	0.0 (0.0)	1.0 (0.7)	
Age, mean (SD^§^)		12.35 ± 5.6	11.31 ± 5.1	0.606^‡^ (CI^††^-3.06-5.15)
Age category, year				0.713^§§^
	1-5	2.0 (11.8)	2.0 (15.4)	
	6-10	5.0 (29.4)	3.0 (23.1)	
	11-15	5.0 (29.4)	6.0 (46.1)	
	>15	5.0 (29.4)	2.0 (15.4)	

Data presented as number and percentage. ^∗^Metabolic, renal, gastrointestinal disorders, and others, ^†^Fisher's Exact test, ^§^standard deviation, ^‡^Student *t* test, ^††^confidence interval, and ^§§^Pearson chi-square.

## Data Availability

Data will be available upon request anytime.
